# Patient perceptions of physician empathy, satisfaction with physician, interpersonal trust, and compliance

**DOI:** 10.5116/ijme.4d00.b701

**Published:** 2010-12-14

**Authors:** Mohammadreza Hojat, Daniel Z. Louis, Kaye Maxwell, Fred Markham, Richard Wender, Joseph S. Gonnella

**Affiliations:** 1 Center for Research in Medical Education and Health Care, Jefferson Medical College of Thomas Jefferson University, USA; 2 Department of Family and Community Medicine, Jefferson Medical College of Thomas Jefferson University, USA

**Keywords:** Keywords: Empathy, patient perception, compliance, satisfaction, trust

## Abstract

**Objectives:**

This study was designed to investigate psychometric properties of the Jefferson Scale of Patient Perceptions of Physician Empathy (JSPPPE), and to examine correlations between its scores and measures of overall satisfaction with physicians, personal trust, and indicators of patient compliance.

Methods: Research participants included 535 out-patients (between 18-75 years old, 66% female). A survey was mailed to participants which included the JSPPPE (5-item), a scale for measuring overall satisfaction with the primary care physician (10-item), and demographic questions. Patients were also asked about compliance with their physician’s recommendation for preventive tests (colonoscopy, mammogram, and PSA for age and gender appropriate patients).

Results: Factor analysis of the JSPPPE resulted in one prominent component. Corrected item-total score correlations ranged from .88 to .94. Correlation between scores of the JSPPPE and scores on the patient satisfaction scale was 0.93. Scores of the JSPPPE were highly correlated with measures of physician-patient trust (*r* >.73). Higher scores of the JSPPPE were significantly associated with physicians’ recommendations for preventive tests (colonoscopy, mammogram, and PSA) and with compliance rates which were > .80). Cronbach’s coefficient alpha for the JSPPPE ranged from .97 to .99 for the total sample and for patients in different gender and age groups.

Conclusions: Empirical evidence supported the psychometrics of the JSPPPE, and confirmed significant links with patients’ satisfaction with their physicians, interpersonal trust, and compliance with physicians’ recommendations. Availability of this psychometrically sound instrument will facilitate empirical research on empathy in patient care in different countries.

## Introduction

Empathy in the context of patient care is defined as a predominantly cognitive attribute that involves an understanding of patients’ experiences, concerns, and perspectives, combined with a capacity to communicate this understanding, and an intention to help.^1^^-^^3^ A key notion in this definition is the communication of understanding which implies that the patient should *perceive* his/her physician’s empathy to better benefit from optimal outcomes.^4^^-^^6^Despite the importance of empathic engagement in patient care^1^^-^^6^ empirical research on its link with patient outcomes is scarce. One reason for scarcity of research on the topic was a lack of a valid and reliable instrument to measure patient perceptions of physician empathy. A few years ago, we developed a brief scale (5-item), the Jefferson Scale of Patient Perceptions of Physician Empathy (JSPPPE), in response to a need for a psychometrically sound instrument for that purpose. Although we havereported some preliminary data in support of the validity and reliability of the scale in small samples,^7^^,^^8^ more definitive evidence is needed to support the psychometrics of the scale in a larger sample to increase the confidence of its users. In addition, it is important to document the relationship between patient perceptions of physician empathy and outcomes, such as satisfaction with physicians, physician-patient interpersonal trust, and compliance, not only to further support the validity of the scale but also as evidence for positive outcomes of empathic engagement in patient care. Therefore, we designed this study to serve the aforementioned purposes.

## Methods

### Participants

Research participants were 535 patients who responded to a mailed survey. These patients were selected based on the following criteria: 1. age between 18 and 75 years at the time of their first visit, 2. had at least two office visits with the physician during the past 36-month time period, 3. spent at least two-thirds of the total office visits with the attending physician identified as the patient’s primary caregiver. The average patients’ age was 54.6 years (*SD*=13.9 yrs); there were 174 men (*n*=33%) and 355 women (*n*=66%) in the sample (six patients did not specify their gender).

### Instruments

The survey instrument included 25 items. The Jefferson Scale of Patient Perceptions of Physician Empathy (JSPPPE) was included in the survey. These items are answered on a 7-point Likert scale (1=Strongly Disagree, 7=Strongly Agree). This brief instrument (5-item) was developed to measure patients’ perceptions of their physician’s empathy. Preliminary data in support of the validity and reliability of this scale have been reported.^7^^,^^8^In addition, a 10-item scale of patient overall satisfaction with primary care physician was included in the survey. Strong evidence in support of validity and reliability of this scale has been reported.^9^ Questions about respondents’ gender, age, education, race, and ethnicity were solicited. Patients over 50 years of age were asked if they had a colonoscopy that was recommended by the physician (named on the survey). For female patients over 50 years of age, we asked if they had a mammogram that was recommended by the physician, and for male patients over 50 years, we asked if they had a prostate-specific antigen **(**PSA) test that was recommended by the physician. Also, questions were included as criterion measures for the validity study to specifically address interpersonal patient-physician trust, (e.g., “I would recommend this doctor to my family and friends.” (1=Strongly Disagree, 7=Strongly Agree).

### Procedures

Subsequent to the approval of the Institutional Review Board of Thomas Jefferson University, we mailed the survey to 2,633 selected patients of the 29 faculty physicians from Jefferson’s Department of Family and Community Medicine.We randomly selected 100 patients for those physicians who had more than 100. The number of selected patients per physician ranged from 46 to 100, with an average of 91 patients per physician.A copy of the survey was mailed with a cover letter explaining the purpose of the study as investigating patient-doctor relationships. Patients were not asked to identify themselves and were assured about the confidentiality of individual responses. The name of the primary care physician was printed on the first page. Patients were asked if the named doctor provided care to them during the past three years, and how often they visited the physician during that time period.Of the total mailed surveys 84 were returned undelivered due to either incorrect addresses or change of addresses. We re-mailed the surveys to those with address changes if the forwarding address was specified on the envelope by the post office. Five surveys were not delivered marked “deceased,” and 32 patients indicated on their returned surveys that the physician named on the survey was not their primary care doctor. We received a total of 535 useable surveys (20% response rate). Patients remained anonymous; thus we could not identify who did or did not respond in order to send a follow-up note to increase the response rate.

### Statistical Analyses

We used principal component factor analysis to examine underlying constructs of the JSPPPE scale. Pearson correlation coefficient was calculated to examine relationships between variables; and *t-*test and analysis of variance were used to test the statistical significance of group differences. When appropriate, effect sizes were calculated to judge the practical importance of the statistically significant findings.^10^^,^^11^ Statistical analyses were performed using SAS version 9.1 for Windows.

## Results

### Underlying Construct of the Scale

Factor analysis of the scores of the JSPPPE item scores resulted in only one prominent factor with an eigenvalue of 4.2, accounting for 84 percent of the variance. The eigenvalues of the other extracted factors were all bellow .29. Factor coefficients are reported in Table 1. Factor coefficients ranged from .84 to .93 indicating that the instrument is a uni-dimensional scale involving only one prominent component.

**Table 1:** Factor Coefficients of the Jefferson Scale of Patient Perceptions of Physician Empathy, Item-Total Score Correlations, and Correlations of Each Item with Scores of Patient Satisfaction and Recommendation (n=535)

Items	Factor Coefficients*	Item-Total Score^✝^	Patient Satisfaction^‡^	Recommendation^¶^
1. My doctor understands my emotions, feelings and concerns	0.93	0.94	0.87	0.80
2. My doctor is an understanding doctor	0.92	0.93	0.95	0.89
3. My doctor seems concerned about me and my family	0.92	0.93	0.87	0.82
4. My doctor asks about what is happening in my daily life	0.88	0.91	0.80	0.73
5. My doctor can view things from my perspective (see things as I see them)	0.84	0.88	0.79	0.74

* Items are reported by descending order of factor coefficients.

✝Correlation between scores of the item and the rest of the scale.

^‡^ Correlation between scores of the item and scores on the Jefferson Scale of Patient Satisfaction.^9^

^¶^ Correlation between scores of the item and responses to this anchor item: “I would recommend my doctor to my family and friends.”

### Concurrent Validity

Correlations between scores on each item of the JSPPPE and measures of patient-physician interpersonal trust were all statistically significant (Table 2) ranging from .73 to .96.

**Table 2:** Concurrent Validity Coefficients of the Jefferson Scale of Patient Perceptions of Physician Empathy and Criterion Measures of Patient-Physician Interpersonal Trust by Patients’ Gender and Age

Criterion Measures	Gender*		Age		Total (n = 535)
	Men (n = 174)	Women (n = 355)	< 56 (n = 266)	≥ 56 (n = 269)	
Patient overall satisfaction with physician^✝^	0.94	0.93	0.96	0.90	0.93
I would recommend my doctor to my family and friends	0.88	0.86	0.91	0.80	0.87
My doctor listens carefully to me	0.88	0.91	0.96	0.84	0.91
My doctor spends sufficient time with me	0.79	0.80	0.85	0.75	0.80
My doctor really cares about me as a person	0.93	0.85	0.89	0.87	0.88
I would like my doctor to be present in any medical emergency situation	0.73	0.78	0.80	0.73	0.77
I am satisfied that my doctor has been taking care of me	0.86	0.86	0.90	0.83	0.87

* Six patients did not specify their gender

^✝^ Scores on the Jefferson Scale of Patient Overall Satisfaction with Primary Care physician ^9^

Correlations between scores of the JSPPPE and scores of overall satisfaction with the physician were greater than .92 for different samples; and correlations between JSPPPE scales and ratings of recommending physicians to family and friends were > .85 (Table 2). Correlations reported in Table 2 provide support for the concurrent validity of the JSPPPE for the total sample as well as for men and women and for younger (< 56 years of age) and older patients (≥ 56 years of age, median split).

### Reliability

We calculated Cronbach’s coefficient alpha which is an indicator of the internal consistency reliability of the instrument (Table 3).

**Table 3:** Descriptive Statistics for the Jefferson Scale of Patient Perceptions of Physician Empathy by Patients’ Gender and Age (n=535)

	Mean	SD	Range	Cronbach’s Alpha
Gender*				
Men (n = 174)	30.7	7.1	5 - 35	0.97
Women (n = 355)	29.2	8.0	5 - 35	0.98
Age^✝^				
< 56 yrs (n = 266)	29.3	8.3	5 - 35	0.99
≥ 56 yrs (n = 269)	29.9	7.3	5 - 35	0.98
Total (n = 535)	29.6	7.8	5 - 35	0.98

*t _(527)_ = 2.17, p < .05 (6 patients did not specify their gender)

^✝^t _(522)_ = .61, p = .95 (nonsignificant)

The reliability coefficients for the total sample and subsamples by gender and age were very large in magnitude (≥.96) indicating that the instrument is highly internally consistent.

### Descriptive Statistics

The means, standard deviations, and ranges of scores of the satisfaction scale for the total sample and for men and women, and younger and older patients are reported in Table 3. The possible range for the scale is 5-35, and the actual range was also 5-35 regardless of patients’ gender and age. The mean score for the total sample was 29.6 (*SD*=7.8). No significant difference was observed between older and younger patients. However men perceived their physicians as more empathic than women, but the difference was not of practical importance (effect size=.20).

### Criterion-Related Validity

#### Colonoscopy

The scores on the JSPPPE were compared for patients over 50 years of age who reported that their doctor did (*n*=333) or did not (*n*=78) recommend colonoscopy. Summary results are reported in Table 4.

**Table 4:** Scores on Jefferson Scale of Patient Perceptions of Physician Empathy and Physicians’ Recommendations for Preventive Tests

Test recommended by physician	M	SD	t
Colonoscopy*			
Yes (n = 333, compliance rate=81%)	30.8	6.5	6.5**
No (n = 78)	24.7	10.6	
Mammogram^✝^			
Yes (n = 256, compliance rate=92%)	30.0	7.2	3.1**
No (n = 58)	26.2	10.3	
PSA^‡^			
Yes (n = 126, compliance rate=90%)	31.3	6.0	3.5**
No (n = 37)	26.5	10.5	

** *p* < 0.01

*Male and female patients over 50 years

^✝^Female patients over 50 years

^‡^ Male patients over 50 years

The mean scores of the JSPPPE was significantly (*p*<.01) higher for those patients whose doctors recommended a colonoscopy screening test (*M*=30.8) than others in the same age group (*M*=24.7). The effect size was .78 indicating that the difference in empathy scores was of practical importance.^10^^,^^11^

It is interesting to note that 81% (*n*=270) of patients who reported that their physician recommended colonoscopy had the procedure done. In contrast, only 27% (*n*=21) of patients who reported that their physicians did not recommend colonoscopy had it done (probably ordered by another physician or by the patient’s own request).

#### Mammogram

The mean score of the JSPPPE for female patients over 50 years of age who reported that their physicians recommended mammogram (*n*=256) was significantly higher than for others in the same gender and age group (*n*=58) whose physician did not recommend the test (*M*=30.0 versus *M*=26.2, respectively, *p* <.01, Table 4). The effect size was .45 indicating that the difference should not be considered negligible. ^10^^,^^11^ The compliance rate was 92% (*n*=236) for the former group. In contrast, only 16% (*n*=9) of patients in the latter group reported having a mammogram.

#### PSA

Male patients over 50 years of age who reported that their physicians recommended the PSA test (*n*=126) obtained a higher mean score on the JSPPPE than their counterparts in the same age group (*n*=37) whose physicians did not recommend the test (*M*=31.3 versus *M*=26.5, respectively, *p* <.01, Table 4). The effect size was .62 indicating that the difference was of practical importance.^10^^,^^11^ The compliance rate was 90% (*n*=114) for the former group, but only 5% (*n*=2) of patients in the latter group reported having a PSA test done.

#### Associations with physician self-reported empathy

Additional analysis was performed to examine the association between scores of the JSPPPE and physicians’ self-report empathy scores. The 29 attending physicians of the participating patients were asked to complete the Jefferson Scale of physician Empathy^1^^,^^12^ for another project on clinical outcomes of physician empathy with diabetic patients.^9^ We classified the physicians into three groups according to the distribution of their empathy scores: high (top third), moderate (middle third), and low (bottom third). We compared the JSPPPE mean scores among these three groups of physicians. The JSPPPE mean scores for the high, moderate, and low empathy scorers were 30.1 (*SD*=7.8), 30.0 (*SD*=7.3), and 28.8 (*SD*=8.3), respectively. As expected, the JSPPPE mean score was lowest for the low empathy scoring physicians and highest for the high empathy scoring physicians, but the results of analysis of variance indicated that the differences did not reach the acceptable level of statistical significance (*F*_(2, 532)_=1.30, *p*=.27).

## Discussion

Findings of this study provide strong evidence in support of the psychometrics of the JSPPPE. Concurrent validity of the scale was supported by significant correlations with scores of the patient satisfaction scale, willingness to recommend the physician to family and friends, and other indicators of interpersonal trust between patients and physicians. These findings are in agreement with those reported by Kim and colleagues in a sample of Korean patients.^13^Criterion-related validity of the scale was supported by higher JSPPPE mean scores among patients whose physicians recommended the preventive tests, and by their high compliance rates. These important findings suggest that physicians’ orientation toward preventive measures can contribute to a more positive perceptions of physician empathy, probably due to patients feeling that their physicians do understand and care about their future health.^4^^,^^14^The findings that patient perception of physician empathy was not a significant predictor of physician’s self reported empathy needs explanation. Consistent with our findings, Kurtz^15^ reported that it was not the therapist’s self-reported empathy, but patient perceived empathic engagement which was significantly associated with clinical outcomes. In one study with 27 internal medicine residents, a nonsignificant correlation of .24 was reported between scores of the JSPPPE and scores of the self-reported Jefferson Scale of Physician Empathy.^7^ In another study with 36 family medicine residents^8^ the correlation between the two aforementioned scales was .48, (*p*<.05). The question of whether these inconsistent findings are due to different views of empathic engagement held by physician and patient, or to other factors remains open for further scrutiny in future research.

### Limitations and Future Research

Limitations of this study include the low response rate and that it took place at a single institution. Both can jeopardize the generalization of the findings. Also, those patients who hold a positive view of their physicians are often more inclined than others to respond to the surveys about their physicians, particularly when the positive view is formed during a relatively long period of being cared by one physician. This may generate a sampling bias that may limit the generalization of the findings. However, the major purpose of this study was to examine the internal relationships between scores of the JSPPPE and selected criterion measures such as satisfaction with physician, trust in physician, and compliance. Future research for generalization of the findings will require a more representative sample from multiple medical centers with a reasonable response rate. Further research is also needed to examine the psychometrics of the JSPPPE for physicians in various specialties and different practice settings.Despite the aforementioned limitations, our findings can add to the confidence of researchers about using the JSPPPE which is supported by strong psychometric evidence as reported in this study. Given the trend toward universal health care, there is a need to translate, validate, and use measure of quality of clinical care in different countries.^16^ The availability of this psychometrically sound instrument can help researchers in those countries to culturally adapt the JSPPPE and conduct cross-cultural studies on patient’s perceptions of physician’s empathic engagement in medical care.

## References

[1] Hojat M. Empathy in patient care: antecedents, development, measurement, and outcomes. New York: Springer, 2007.

[2] Hojat M Vergare MJ Maxwell K Brainard G Herrine SK Isenberg GA Veloski J Gonnella JS. The devil is in the third year: a longitudinal study of erosion of empathy in medical school. Acad Med. 2009;84:1182-1191 19707055 10.1097/ACM.0b013e3181b17e55

[3] Hojat M. Ten approaches for enhancing empathy in health and human services culture. J Health Hum Serv Adm. 2009;31:412-450 19385420

[4] Squier RW. A model of empathic understanding and adherence to treatment regimens in practitioner-patient relationships. Soc Sci Med. 1990;30:325-339 2408150 10.1016/0277-9536(90)90188-x

[5] Free NK Green BL Grace MC Chernus LA Whitman RM. Empathy and outcome in brief focal dynamic therapy. Am J Psychiatry. 1985;142:917-921 4025586 10.1176/ajp.142.8.917

[6] Spiro HM, McCrea Curren MGM, Peschel E, St. James D. Empathy and the practice of medicine: beyond pills and the scalpel. New Haven: Yale University Press, 1993.

[7] Kane GC Gotto JL Mangione S West S Hojat M. The Jefferson scale of patient’s perceptions of physician empathy: preliminary psychometric data. Croat Med J. 2007;48:81-86 17309143 PMC2080494

[8] Glaser K Markham FW Adler HM McManus PR Hojat M. Relationship between scores on the Jefferson Scale of Physician Empathy, patient perceptions of physician empathy, and humanistic approaches to patient care: a validity study. Med Sci Monit. 2007;13:291-294 17599021

[9] Hojat M Louis D Maxwell K Markham F Wender R Gonnella JS. Psychometrics of a brief instrument to measure patient’s overall satisfaction with primary care physicians. Fam Med, in press 21656396

[10] Cohen J. Statistical power analysis for behavioral sciences. Hillsdale, NJ, Erlbaum, 1987.

[11] Hojat M Xu G. A visitor’s guide to effect size: Statistical versus practical (clinical) importance of research findings. Adv Health Sci Educ Theory Pract. 2004;9:241-249 15316274 10.1023/B:AHSE.0000038173.00909.f6

[12] Hojat M Gonnella JS Nasca TJ Mangione S Vergare M Magee M. Physician empathy: definition, components, measurement and relationship to gender and specialty. Am J Psychiatry. 2002;159:1563-1569 12202278 10.1176/appi.ajp.159.9.1563

[13] Kim SS Kaplowitz S Johnston MV. The effect of physician empathy on patient satisfaction and compliance. Eval Health Prof. 2004;27:237-251 15312283 10.1177/0163278704267037

[14] DiMatteo MR, DiNicola DD. Achieving patient compliance: the psychology of the medical practitioner’s role. New York: Pergamon Press, 1982.

[15] Kurtz RR. Grummon DL. Different approaches to the measurement of therapist empathy and their relationship to therapy outcomes. J Consult Clin Psychol. 1972;39:106-115 5045268 10.1037/h0033190

[16] Wild D Eremenco S Mear I Martin M Houchin C Multinational trail_ Recommendations on the translations required, approaches to using the same language in different countries, and the approaches to support pooling the data: the ISPOR patient-record outcomes translation and linguistic validation good research practices task force report. Value Health. 2009;12:430-440 19138309 10.1111/j.1524-4733.2008.00471.x

